# Transactions of California State Medical Society

**Published:** 1892-08

**Authors:** 


### TRANSACTIONS OF THE FIFTEENTH ANNUAL SESSION OF THE CALIFORNIA STATE HOMŒOPATHIC MEDICAL SOCIETY. Held at San Francisco, Cal., May 13th and 14th, 1891. Vol. II. San Francisco, 1892.

This is a pleasant-looking little volume of some two hundred pages, and does credit to our brethren in California. High potencies do not seem to be much thought of by most of the writers, as shown by the discussions following the reports of cures made with them.

The most interesting reading to us was a paper sent to the Society by Dr. S. Lilienthal, entitled, "Homœopathic Therapeutics." On account of sickness he could not personally attend, and the paper was read by his son, Dr. J. E. Lilienthal. In this paper he gives the Society some good advice, and urges it to be true to pure Homœopathy. He tells the Society of a physician who once told him that before starting out on his daily rounds he prayerfully read a part of a lecture contained in that great work, *Homœopathy, the Science of Therapeutics*, and that he felt strengthened by its perusal, and that his patients got the full benefit of this pious act. Lilienthal adds that it is his fervent prayer that the members of the Society may follow the example of this physician, because of reading this great work of Carroll Dunham they would know all about homœopathic therapeutics.

Good advice! Accept and practice it, all ye followers of Hahnemann, and do not let this bacteriological craze turn your heads, but cling now and forever to the strict inductive method of the master. W. S.

